# Rare cause of complicated Meckel’s with Schistosoma infection: An unusual cause of acute intestinal obstruction in adults

**DOI:** 10.1016/j.ijscr.2019.11.039

**Published:** 2019-12-19

**Authors:** Fahad Almadi, Emad Aljohani

**Affiliations:** a Department of Surgery, King Saud Medical City, Riyadh, Saudi Arabia; b Department of Surgery, College of Medicine, Prince Sattam Bin Abdulaziz University, Al-kharj, Saudi Arabia

**Keywords:** CT, computed tomography, DRE, digital rectal exam, SBO, small bowel obstruction, GIA, gastrointestinal anastomosis, MD, Meckel’s diverticulum, Acute bowel obstruction, Schistosoma, Meckel’s diverticulum

## Abstract

**Introduction:**

Small bowel obstruction (SBO) is a common surgical condition, while Meckel’s diverticulum (MD) is the most common congenital anomaly of the gastrointestinal (GI) tract, for which differential diagnosis is considered among children. Intestinal parasitosis refers to a group of disease caused by one or more species of protozoa, cestodes, trematodes, and nematodes. Intestinal parasitic infections (IPIs) caused by pathogenic helminths and protozoan species are endemic throughout the world. The intestine is frequently involved during Schistosoma infection, especially with *Schistosoma mansoni*.

**Presentation of case:**

A 41 years old Egyptian male with an unremarkable past medical or surgical history presented to the emergency department with two days history of abdominal pain associated with nausea, vomiting, and abdominal distention. The pain was colicky and started gradually without any bowel motion or flatus for two days. Patient had tachycardia with other vitals within the normal limit. The abdomen was found to be distended and tender all over with an empty rectum by digital rectal examination (DRE). Upright chest and abdominal X-rays showed multiple air fluid levels. CT abdomen showed dilated proximal small bowel loop and a picture of SBO at distal ileal loops. The patient was resuscitated with IV fluids and shifted to the operating room. Laparoscopic exploration showed dilated small bowel loops with collapsed distal ileum, and an inflammatory band was found 20 cm from ileocecal junction, originating from a small bowel diverticulum. The inflammatory band was released, and the diverticulum was resected at the base by gastrointestinal anastomosis (GIA). Postoperatively, the patient significantly improved, passed bowel motion and was discharged for home. The histopathology report showed MD with Schistosoma infection. The patient was then referred to the infectious disease department and praziquantel, a drug of choice for schistosomiasis, effective against all *Schistosoma species* was initiated.

**Discussion:**

Schistosomiasis is a parasitic disease that is transmitted through contact with contaminated water sources. Our patient had complete SBO with significant symptoms. He was treated with the surgical approach with diverticulum resection. Intestinal schistosomiasis was found on histopathology testing. In summary, we report a rare presentation of acute small bowel obstruction with an inflammatory band around MD harboring parasitic infection.

**Conclusion:**

Diagnostic laparoscopy should be considered for all patients having virgin abdomen presenting with complete bowel obstruction. To the best of our knowledge from the literature review, this is the first case to be reported as acute SBO secondary to MD harbouring *Schistosoma mansoni* infection.

## Highlights

• Small bowel obstruction is a common surgical condition with diversity of causes.
• Meckel’s diverticulum is the most common congenital anomaly causing bowel obstruction.
• Complicated Meckel’s diverticulum in adults infected with parasitic infection is rare.

## 1 Introduction

Small bowel obstruction (SBO) is a common surgical condition, accounting for 50 % of emergency laparotomies each year in the UK and over 300,000 admissions in the United States of America (USA), annually. The condition is associated with a mortality rate of approximately 10 %, and high morbidity rates among survivors. The causes of SBO are diverse; and surgery, may be indicated, depending on the suspected cause and patient factors [1]. Meckel’s diverticulum (MD) is the most common congenital anomaly of the gastrointestinal (GI) tract, which results from incomplete obliteration of the vitelline duct, leading to the formation of a true diverticulum of the small intestine [2]. Most patients with MD remain asymptomatic lifelong, but children with the disease may develop symptoms of massive GI bleeding and rare complications including intestinal obstruction, intussusception, hernia, inflammation, and perforation [3]. Intestinal parasitosis refers to a group of diseases caused by one or more species of protozoa, cestodes, trematodes, and nematodes. Intestinal parasitic infections (IPIs) caused by pathogenic helminths and protozoan species are endemic throughout the world [4]. The intestine is frequently involved during Schistosoma infection, especially with *Schistosoma mansoni*. We report a rare presentation of acute SBO with an inflammatory band around MD. The work has been reported in line with the Consensus Surgical CAse REport (SCARE) criteria [5].

## 2 Case presentation

**Fig. 1:**
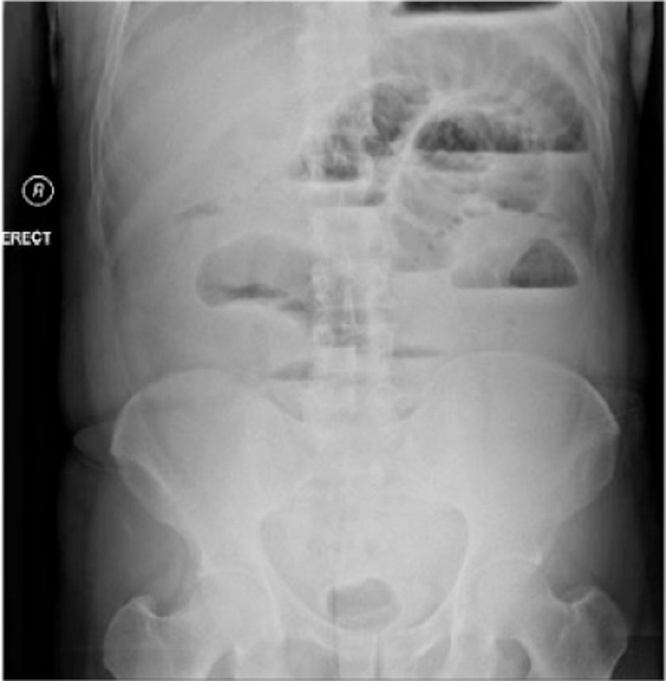
abdomen x ray, showed multiple air fluid level.

**Fig. 2:**
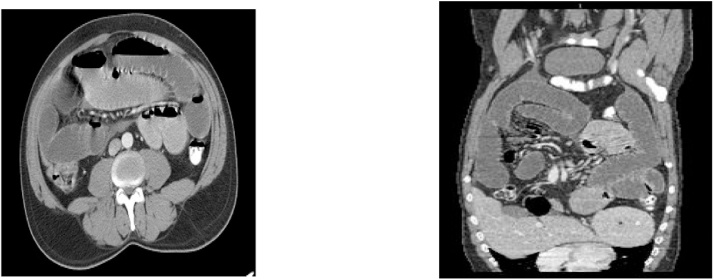
CT abdomen showing Small bowel complete obstruction with transitional zone seen distal ilealloops.

**Fig. 3:**
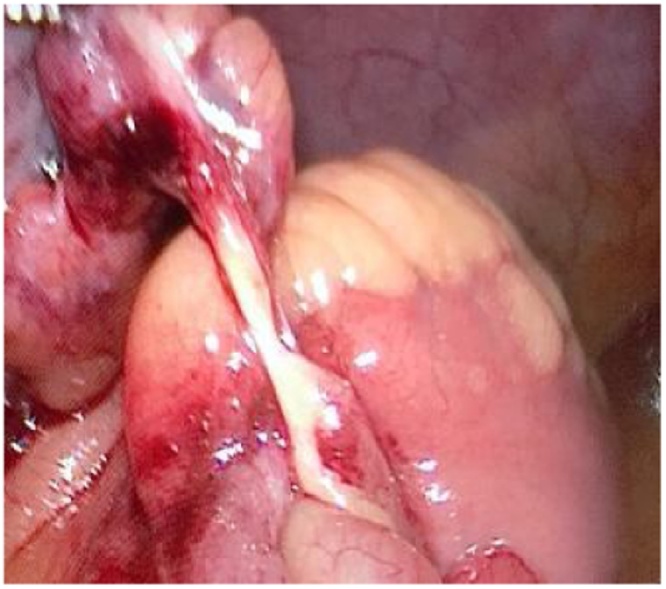
laparoscopic view showing a band 20 cm from ileocecal valve originating from a small bowel diverticulum.

A 41 years old Egyptian male with an unremarkable past medical or surgical history presented to the King Saud medical city emergency department, with two days history of abdominal pain, associated with nausea, vomiting, and abdominal distention. The pain was colicky and started gradually without any bowel motion or flatus for two days. The drug and family history was unremarkable. Patient had tachycardia, with normal blood pressure and oxygen saturation of 100 % in room air. The abdomen was found to be distended and tender all over with an empty rectum by digital rectal examination (DRE). Upright chest and abdominal X-ray showed multiple air fluid levels with no air under the diaphragm (Fig. 1). CT abdomen showed a dilated proximal small bowel loop and a picture of SBO at distal ileal loops (Fig. 2). The patient resuscitated with intravenous (IV) fluids and kept nil per os (NPO). A nasogastric tube was inserted, and the patient was shifted to the operating room. Laparoscopic exploration showed dilated small bowel loops with collapsed distal ileum and an inflammatory band was found 20 cm from ileocecal junction originating from a small bowel diverticulum with no palpable intraluminal masses (Fig. 3). The inflammatory band was present at the distal part of the diverticulum and was adhered to a small bowel segment leading to obstruction. The band was released, and wedge resection of the diverticulum was performed at its base by gastrointestinal anastomosis (GIA) on healthy tissue, about 2 cm from the inflamed segment, which is a standard surgical approach for MD. The postoperative period was uneventful. The patient was initially kept at regular surgical floor bed and then discharged on the second postoperative day, when he passed good bowel motion. The histopathology result on follow up visit showed MD with Schistosoma infection, most likely by *Schistosoma mansoni*. The patient was referred afterwards to the infectious disease department for initiation of praziquantel, which is the drug of choice for schistosomiasis and is effective against all *Schistosoma species*. This is an extremely rare case of acute SBO. It demonstrates the importance of diagnostic laparoscopy in all patients with virgin abdomen who present with acute bowel obstruction.

## 3 Discussion

MD originates from incomplete obliteration of the vitelline duct, which occurs around the fifth week of gestation. The complications of MD are greatest in the first two years of life. Haemorrhage, secondary to peptic ulceration, is the most frequent manifestation. Next, is the frequency of intestinal obstruction and intussusception being the leading cause. Intestinal obstruction accounts for 30%–56% of symptoms of MD. Commonly documented mechanisms of obstruction are volvulus, intussusception, bands, Littre's hernia, internal hernias, and strictures [6,7].

Schistosomiasis is a parasitic disease transmitted through contact with contaminated water sources. It is related to bad personal hygiene and environmental sanitation. This explains the dominance of this disease in the endemic areas within the developing countries. The disease is endemic in Sudan and Egypt. *Schistosoma mansoni* and *Schistosoma japonica* represent the types that infest the intestine [8].

Our patient had complete SBO with significant symptoms. He was treated with laparoscopic exploration with band release and diverticulum resection. Intestinal schistosomiasis was found on histopathology. He was treated with praziquantel after surgery, which is the drug of choice for schistosomiasis and is effective against all *Schistosoma species*. In summary, we report a rare presentation of acute small bowel obstruction with an inflammatory band around MD harbouring parasitic infection. To the best of our knowledge from the literature review, this is the first case report to be reported on acute SBO secondary to MD harbouring *Schistosoma mansoni* infection.

## Sources of funding

No fund to my research to be disclosed.

## Ethical approval

This is case report study and ethical approval not required.

## Consent

Written informed consent was obtained from the patient for publication of this case report and accompanying images. A copy of the written consent is available for review by the Editor-in-Chief of this journal on request.

## Author contribution

Dr.Emad Aljohani (case description and discussion).

Dr.Fahad Almadi (literature review and collected the data from the patient file).

## Registration of research studies

5143.

## Guarantor

Dr. Emad Aljohani.

## Provenance and peer review

Not commissioned, externally peer-reviewed.

## Declaration of Competing Interest

No conflict of interest.
